# Na^+^/K^+^-ATPase, cardiac glycosides, and tumor immunity

**DOI:** 10.3389/fchem.2026.1811060

**Published:** 2026-04-22

**Authors:** Yulin Ren, Jianhua Yu, Xiaolin Cheng, A. Douglas Kinghorn

**Affiliations:** 1 Division of Medicinal Chemistry and Pharmacognosy, College of Pharmacy, The Ohio State University, Columbus, OH, United States; 2 Division of Hematology and Oncology, Department of Medicine, School of Medicine, University of California Irvine, Irvine, CA, United States; 3 The Clemons Family Center for Transformative Cancer Research, Chao Family Comprehensive Cancer Center, University of California Irvine, Irvine, CA, United States

**Keywords:** cardiac glycosides, H+-ATPase, ion channels, Na+/K+-ATPase, tumor immunity

## Abstract

Tumor immunity arises from the coordinated action of innate and adaptive immune systems but is hindered by immune escape within the immunosuppressive tumor microenvironment (TME), for which ion channels and ion pumps have proved to be important. These proteins regulate a wide range of cellular processes to influence cancer progression and immune cell functions, of which ion channels maintain intracellular ion concentrations, cytosolic pH, and cell volume and functions and are essential for cancer development and immune regulation. Ion pumps correlate closely with ion channels and show an important effect on tumor immunity. Of these, H^+^-ATPases, especially vacuolar H^+^-ATPase (V-ATPase), play critical roles in cancer progression, metastasis, and immune evasion, while Na^+^/K^+^-ATPase (NKA) interacts with ion channels and H^+^-ATPase and hence contributes to antitumor immune responses. Thus, several cardiac glycoside inhibitors have been reported to exert potent antitumor and immunomodulatory activities. In the present perspective article, the interconnections among NKA, ion channels, H^+^-ATPases, and immune responses are addressed, with the potential activities of cardiac glycosides on tumor immunity discussed.

## Introduction

Neoplastic cells, the precursors of cancer, are subject to constant interaction with the immune system, where immune surveillance is essential for recognizing and eliminating potentially malignant cells. The antitumor immune responses initiate from the innate immune system [including natural killer (NK) cells and macrophages] that is alerted by a growing tumor, but surviving cancer cells eventually can evolve mechanisms to evade immune surveillance. Thus, cancer immunoediting that consists of immunosurveillance and tumor progression working with each other through three phases, elimination, equilibrium, and escape, is crucial for understanding cancer development and cancer immunotherapy (Sengupta et al., 2010).

Tumor immunity (immunity against tumors) refers to the body’s defense against cancer, with the innate and adaptive immune systems being involved, including all types of immune cells, such as T cells, B cells, NK cells, macrophages, dendritic cells (DC), granulocytes, and mast cells. However, immune cells in the surroundings of tumors (the tumor microenvironment, TME) interact to regulate tumor growth, and this leads to tumor immune evasion through immunoediting, including reducing antigen presentation and activating the immune checkpoint and recruiting the immunosuppressive cells to suppress immune cell activity. In addition, a complex network mediated by cancer cells and the associated milieu can blunt immune reactivity to silence adaptive immunity against these cancer cells. For example, tumor exosomes, the endosomal-derived nanovesicles released by cancer cells, can convey information between cancer cells and their neighboring immune cells to contribute to cancer progression (Filipazzi et al., 2012).

Tumor immune escape represents a major problem in tumor immunity. This phenomenon is attributed to the inability of immune cells to conduct their functions and their antitumor response, in which cancer stem cells (CSCs) play a key role (Tsuchiya and Shiota, 2021; Whiteside, 2009). To escape from immune detection, tumors either disguise themselves or are protected from immune attack with an immunosuppressive TME, on which the immune system focuses on improving an antitumor response (Yang et al., 2024). Thus, the TME has been regarded as a crucial target for the development of cancer immunotherapy (Sinha et al., 2025), and, as an important component of the TME, CSCs have attracted wide interest (Mahanti and Bhattacharyya, 2024). In the TME, T cell infiltration functions as an effective tumor destructor to recognize and attack cancer cells and thus plays a critical role in tumor immunity. Accordingly, chimeric antigen receptor (CAR)-T cell therapy has been developed successfully for the treatment of certain types of leukemia. However, in the immunosuppressive TME, T cells, including CAR-T cells, can be exhausted, which may limit clinical outcomes of CAR-T cell immunotherapy. Fortunately, a combination therapeutic strategy has proved to be able to improve the efficacy and safety of T cell-based immunotherapy, and this could benefit from a further understanding of the TME, tumor immune evasion, and the immune system (Ma et al., 2025).

Ion channels and pumps regulate cellular membrane potential, ion homeostasis, and electric signaling. They can also form macromolecular complexes with signaling molecules and thus are important for cell proliferation, migration, apoptosis and differentiation, as well as for cancer progression (Litan and Langhans, 2015). The ion channels also function as enzymes and play important roles in regulating innate and adaptive immunity (Cahalan, 2001; Vaeth and Feske, 2018). Thus, ion channels and pumps could show promise to alter the TME immune evasion mechanisms to support tumor immunity, as discussed in the following paragraphs.

## Ion channels and tumor immunity

Ion channels regulate various cellular processes and are essential for immune cell function, and thus they offer a target for cancer immunotherapy. These channels govern the intracellular ion concentration, cytosolic pH, and cell volume and hence are essential for cell proliferation and play an important role in the development of cancer (Bates, 2015; Kunzelmann, 2005; Leanza et al., 2013). For example, the mechanosensitive ion channels that are involved in the Ca^2+^-signaling of the tumor and stroma cells are invaluable for these cells to respond to mechanical stimuli, including cell migration, proliferation, tissue invasion, and/or tumor fibrosis (Pethő et al., 2019). The intermediate-conductance calcium-activated potassium 3.1 (KCa3.1) channels regulate the membrane potential and maintain calcium homeostasis to show diverse roles in cancer cells (Van and Nam, 2024). In turn, the acid-sensing ion channels (ASICs), the proton-sensitive receptors, play an important role in cancer progression probably by targeting Ca^2+^-signaling, through the effects of pH alterations on the function and activity of cellular proteins and the acidic TME (Gründer et al., 2024). Moreover, transmembrane protein 16 (TMEM16), the calcium-activated chloride channel (CACC), and potassium channels associate with tumorigenesis and metastasis to function potentially as cancer biomarkers and a therapeutic target (Li et al., 2023a; Li et al., 2023b). These, along with other channels, exhibit some potential use in cancer diagnosis and treatment (Lastraioli et al., 2015; Prevarskaya et al., 2018), especially for glioblastoma multiforme (GBM), in which ion channels show extensive remodeling (Kulkarni et al., 2025).

In addition, ion channels, including Ca^2+^, K^+^, Na^+^, Zn^2+^, H^+^, and Cl^−^ channels, are involved in the regulation of immune cell activity and the development of tumors, and appear to play a significant role in the interaction between the immune system and cancer. Of these, calcium (Ca^2+^) channels play a crucial role in producing cytotoxic chemicals by immune cells, and potassium (K^+^) channels are essential for T cell activation and proliferation, while chloride (Cl^−^) channels are involved in immune cell infiltration and invasion into malignancies. These channels also act on the TME to show their potential for the diagnosis and treatment of cancer (Erdogan et al., 2023).

The immune response is involved in motility and cellular interactions, for which T cells contact antigen-bearing DCs to initiate T cell signaling to lead to a series of Ca^2+^ spikes. A rapid rise of cytosolic free Ca^2+^ can trigger T-cell activation, while the Ca^2+^ signal controls T cell development and immunity and acts with other signaling pathways for gene expression, cytokine secretion, and cell proliferation and thus plays a critical role in the immune response (Germain et al., 2012). In human leukemic Jurkat T cells, Ca^2+^ oscillations depend upon Ca^2+^ influx across the plasma membrane, in which the membrane Ca^2+^ conductance plays a significant role in activation of T cells (Lewis and Cahalan, 1989). In addition, Ca^2+^ influx through plasma membrane store-operated Ca^2+^ (SOC) channels, the major pathway for Ca^2+^ signaling, is triggered by depletion of the endoplasmic reticulum (ER) Ca^2+^ store, for which stromal interaction molecule 1 (STIM1) acts as a calcium sensor for SOC entry (SOCE) (Cahalan, 2009; Prakriya and Lewis, 2015). K^+^ channels help regulate Ca^2+^ signaling, and the ATP-sensitive K^+^ (K_ATP_) channel subunits are highly expressed in NK and NKT cells and are upregulated in CD8^+^ T cells and macrophages after infection, and thus, these K^+^ channels play some role in immunity (Feske et al., 2024).

The immune system consists of immune cells that are exposed to varying mechanical stimulation, in which ion channels regulate the cell functions and hence play pivotal roles (Atcha et al., 2021a). These channels direct Ca^2+^ signaling to trigger T-lymphocyte activation (Cahalan and Chandy, 2009; Dong et al., 2017), of which Piezo-type mechanosensitive ion channel component 1 (Piezo1) channels, non-selective cation channels to conduct both mono- and divalent cations, including Na^+^, K^+^, and Ca^2+^, restrain regulatory T (T_reg_) cells but do not affect effector T cell functions (Jairaman et al., 2021). Voltage-gated Ca^2+^ channels (VGCCs) that are composed of pore-forming α1 (Cavα1) and several auxiliary β (Cavβ), α2δ (Cavα2δ)-, and γ (Cavγ)-subunits do not contribute to Ca^2+^ signaling in T cells (Cahalan, 2022), while their Cavβ1 subunits regulate T cell functions in a VGCC-independent manner (Erdogmus et al., 2022). However, as the main source for Ca^2+^ influx, Ca^2+^ release-activated Ca^2+^ (CRAC) channels are required for T cell functions (Vaeth et al., 2020).

The volume-regulated anion channels (VRACs) regulate cell volume through moving Cl^−^ and organic osmolytes across the membrane. The essential component, leucine-rich repeat-containing protein 8 (LRRC8), has been reported as a negative regulator of T cell function (Concepcion et al., 2022). Moreover, ion channels are involved in maturation, migration, and cytokine synthesis and secretion of dendritic cells (DCs), the antigen-presenting cells (APCs) that are crucial for immune responses (Vandier and Velge-Roussel, 2018; Zhou et al., 2025). These channels also play a critical role in the regulation of function of macrophages (Li et al., 2025), in which Piezo1 modulates polarization responses and is involved in stimulation of Ca^2+^ signaling by extracellular oxidized low-density lipoproteins (Atcha et al., 2021b; Atcha et al., 2024).

Ion channels contribute to the regulation of immune cell development and innate and adaptive immune responses and thus function potentially as molecular targets for therapeutic strategies against immune-related diseases (Feske et al., 2015; Zhang et al., 2023). These channels regulate the Ca^2+^ influx and its downstream signaling pathways and the immune system to recognize and eliminate cancer cells (Bose et al., 2015), and thus they have shown promise in cancer immunotherapy (Bose et al., 2015; Panyi et al., 2014). Importantly, ion channels support tumor immunity through their effects on the TME, in which metal ions support regulation of the immune cell functions, activation of the innate immune system, and induction of cancer cell mortality (Gao et al., 2024). Interestingly, necrotic cancer cells were found to release intracellular K^+^ into the extracellular fluid of tumors, which suppressed T cell effector function and the T cell receptor (TCR)-driven Akt-mammalian target of rapamycin (mTOR) phosphorylation (Eil et al., 2016). Thus, K^+^ channels are critical in tumor immunity. These channels, along with other channels, play an important role in immune responses and thus show potential benefits to cancer immunotherapy (Chirra et al., 2022; Gentile et al., 2025).

## H^+^-ATPase and tumor immunity

The TME is the ecosystem surrounding a tumor composed of malignant and non-malignant cells (including immune cells), the extracellular matrix, and blood vessels, which interact to evade tumor immunity to support cancer cell survival and tumor growth (Balkwill et al., 2012). Deregulated energy metabolism, insufficient perfusion, and uncontrolled proliferation of cancer cells lead to acidosis of the TME. This acidic condition can cause double-stranded DNA breaks and inhibits recovery from sublethal DNA damage induced by other stressors to increase the risk of a transition to cancer. In addition, the acidic pH contributes to the maintenance of stemness to support the CSC phenotype and inhibits antitumor immune responses to protect tumor growth against immune-mediated destruction (Boedtkjer and Pedersen, 2020). Also, such microenvironmental acidity affects tumor immune surveillance and contributes to immune escape and cancer progression and hence has been regarded as a potential target for cancer immunotherapy (Huber et al., 2017). In this regard, vacuolar-type (V-type) H^+^-ATPases (V-ATPases) pump protons across the plasma membrane and play key roles in the acidification of the TME and thus show some effects on tumor immunity (De Milito and Fais, 2005).

V-ATPase acidifies a wide array of intracellular organelles, regulates the activity of various signaling pathways, and hence is involved in membrane traffic and protein degradation to influence human health (Eaton et al., 2021). In cancer cells, V-ATPase is involved in the regulation of cellular signaling, cell apoptosis, autophagy, and survival, as well as cell invasion, migration, and metastasis (Chen et al., 2022; Stransky et al., 2016). The acidic environment caused by V-ATPase can also aid the development of drug resistance, and thus it has been used as a target for the development of anticancer agents (Fais et al., 2007). In addition, V-ATPase is involved in cytotoxic T lymphocytes (CTLs), extracellular vesicle endocytosis, the innate immune response, and phagocytosis through association with spleen tyrosine kinase, interferon (IFN), and STING (Ren et al., 2025). It is highly expressed in metastatic breast cancer cells, for which tumor-associated neutrophils (TAN) are associated with increased angiogenesis and metastasis. A cleaved peptide from V-ATPase a2 isoform (a2V), a2NTD, was found to correlate with neutrophil recruitment, blood vessel density, and innate immunity, and treatment with a2NTD increased the secretion of interleukin (IL)-1RA, IL-10, C-C chemokine ligand (CCL)-2 and IL-6 by neutrophils (Ibrahim et al., 2015). Also, the a3-subunit of V-ATPase was found to be responsible for the acidification of cytotoxic granules to contribute to their maturation and efficient transport to the immune synapse, where CTLs release cytotoxic proteins to lead to tumor immunity (Chitirala et al., 2020).

The immune system is regulated by some mechanisms that converge at lysosomes, of which the catabolic activity is correlated with acidic pH levels and thus is governed by V-ATPase. As a result, inactivation of V-ATPase was found to inhibit lysosomal acidification to lead to lysosomal dysfunction, which, in turn, enhances innate immunity, through promoting the nuclear localization of Defective proVEntriculus 1 (DVE-1) and activating the unfolded protein response (UPR_mt_). These indicate that V-ATPase links lysosome, DVE-1, and UPRmt to control immune reactions (Pu and Qi, 2024). Hence, H^+^-ATPases, particularly, V-ATPases, affect tumor cell behavior and TME and their impact on immune responses, as well as cancer progression, metastasis, and immune evasion. These enzymes play a multifaceted role in tumor immunity and are emerging as potential targets for cancer immunotherapy.

## Connections among Na^+^/K^+^-ATPase, ion channels, and H^+^-ATPase

The enzyme Na^+^/K^+^-ATPase (NKA) was proposed by Prof. Jens C. Skou in 1957 and confirmed later from its crystal structure. It is an energy-transducing transmembrane ion pump with enzymatic activity and is involved in the transport of Na^+^ ions across the cell membrane. NKA functions as an ion pump to conserve different transmembrane Na^+^ and K^+^ ion concentrations to restore Na^+^ and K^+^ electrochemical gradients across the plasma membrane through ion-coupled transport processes. These gradients represent an energy source to produce a driving force for the secondary transport of metabolic substrates and for the creation of a membrane potential, the basis for the function of all excitable cells (Morth et al., 2011; Skou, 1998).

**FIGURE 1:**
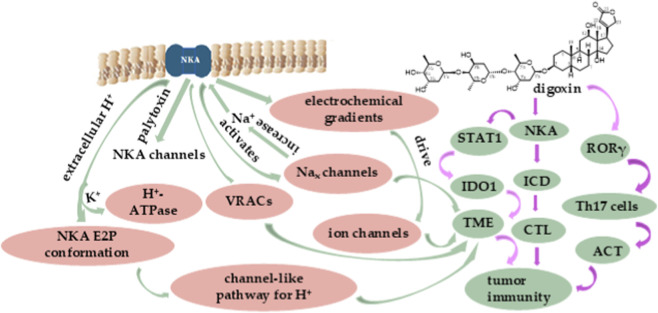
Major connections between tumor immunity and NKA, ion channels, and V-ATPase (left) or digoxin (right).

There are close correlations between ion pumps and ion channels (Figure 1). Ions move down their concentration and electrical gradients across cell membranes through ion channels, while ion pumps use energy provided by the hydrolysis of ATP to drive ions against these gradients. In addition, the ion pumps build ion concentration gradients across the membrane to power ionotropic signaling through ion channels and the uptake and extrusion of other solutes through secondary transporters and facilitators (Morth et al., 2011).

Both ion channels and ion pumps transport ions selectively, which is crucial to the ion-transport proteins. Na^+^ and K^+^ are the most abundant cations in biological systems, and their gradients across the cell membrane provide the energy source for action potentials from opening Na^+^ and K^+^ channels (Gouaux and MacKinnon, 2005). Interestingly, the Na_x_ channels that are responsible for monitoring the Na^+^-levels interact with the NKA α subunit directly to affect potentially its molecular properties. These channels are sensitive to Na^+^ concentrations and open upon increased levels of extracellular Na^+^, while the increased Na^+^ can activate NKA (Reinhard et al., 2013). When treated with the marine natural product palytoxin, the binding sites of NKA are accessible simultaneously from both sides of the membrane, and NKA is transformed into ion channels, which support a rapid Na^+^ flow through the resultant palytoxin-bound NKA-channels (Takeuchi et al., 2008). Also, the crosstalk between NKA and the volume-regulated anion channels (VRACs) has been demonstrated. The non-pumping NKA was involved in VRAC activation induced by the cardiac glycoside ouabain, for which leucine-rich repeat containing 8 family, member A (LRRC8A, also known as SWELL1), an essential component of VRAC, was found to co-immunoprecipitate with the α1 subunit of NKA (α1NaK) in the membrane microdomains (Fujii et al., 2018) (Figure 1).

Also, a close correlation between NKA and H^+^-ATPase has been observed. Acidification of the extracellular solution increases the opening probability of the intracellular gate of NKA trapped in the E2P conformation, which provides a channel-like pathway for both H^+^ and Na^+^. In the absence of extracellular Na^+^ and K^+^. NKA carries an acidic pH-activated and ouabain-sensitive “leak” current, and extracellular H^+^ and Na^+^ share a high field access channel between the extracellular solution and the Na^+^ III binding site of NKA (Li et al., 2006). Moreover, K^+^ binds to H^+^-ATPase at a site involving Asp617, which induces dephosphorylation of the phosphorylated E1P of H^+^-ATPase reaction cycle and protects the H^+^-ATPase against thermal inactivation, while D684N H^+^-ATPase is hypersensitive to K^+^ (Buch-Pedersen et al., 2006) (Figure 1).

## Effects of Na^+^/K^+^-ATPase and its inhibitors, cardiac glycosides, on tumor immunity

As discussed above, NKA correlates with ion channels and H^+^-ATPase and thus shows potential effects on tumor immunity (Figure 1) (Buch-Pedersen et al., 2006; Li et al., 2006), while it restores Na^+^ and K^+^ electrochemical gradients across the plasma membrane and regulates intracellular calcium to support cellular processes (Clausen et al., 2017; Cui and Xie, 2017). Also, NKA is a signal transducer and interacts directly with Src to form a functional signaling complex (Tian et al., 2006). Importantly, NKA is overexpressed or dysregulated in various cancers, with the altered activity contributing to cancer cell survival, proliferation, migration, and metastasis, and hence it has been regarded as promising anticancer target (Garcia et al., 2015; Lu et al., 2021; Mijatovic et al., 2008). Thus, inhibition of NKA can induce apoptosis and autophagy in cancer cells (Goncalves-de-Albuquerque et al., 2017), as well as decreases of cell adhesion, motility, and migration (da Silva et al., 2021), for which the β-subunit of NKA is required (Rajasekaran et al., 2001).

Disrupted Na^+^ homeostasis in cancer leads to raised Na^+^ levels in solid tumors, which, in turn, affect cell volume and cell metabolism, indicating that NKA plays a key role in the TME (Leslie et al., 2019). Recently, endogenous ouabain (EO) was found to increase the transcription of programed death ligand 1 (PD-L1), a protein to break T cells to support TME, but its receptor, NKA a1, interacts with PD-L1 to trigger endocytic degradation. Thus, the NKA/EO signaling pathway could promote immune escape in non-small cell lung cancer (NSCLC) by regulating the programmed death receptor 1 (PD-1)/PD-L1 axis. These observations indicate that PD-1/PD-L1-based immunotherapy may be improved by targeting NKA/EO-induced immunosuppression (Yang et al., 2023).

Several well-known cardiac glycoside NKA inhibitors, as represented by digoxin, oleandrin, and ouabain, are hormone-like immunoregulators. These agents show different bioactivities and have contributed to the treatment of cardiovascular diseases and also have potential for use in the chemotherapy of cancer and certain infections. For example, several cardiac glycosides suppress or induce autophagy and target the DNA damage response and other signaling pathways to mediate their potential antitumor activity. They also induce immunogenic cell death (ICD) to stimulate anticancer immune responses (Bejček et al., 2021; Kepp et al., 2012; Newman et al., 2008). Autophagy can trigger ICD, which is characterized as the release of damage-associated molecular patterns (DAMPs) that can be recognized by the immune system, and thus, as autophagy modulators, cardiac glycosides could be supportive of the effective treatment of cancer (Škubník et al., 2021a).

Cardiac glycosides mediate their potential anticancer and immunogenic activities by targeting NKA, while they stimulate anticancer immune responses through the induction of ICD (Schneider et al., 2017). Also, these compounds inhibit nuclear factor κ-light-chain-enhancer of activated B cells (NF-κB) to show effects on the regulation of immune response genes and suppress the activity of T-helper (Th) cells to modulate the cytokine levels. Importantly, they also induce immunological memory and thus show potential for the development of cancer vaccines (Škubník et al., 2021b).

In addition, the TME mediates its immunosuppression through immune checkpoint proteins, including indoleamine-pyrrole 2′,3′-dioxygenase 1 (IDO1). In A549 lung and MDA-MB-231 breast human cancer cells, the cardiac glycosides, digoxin and ouabain, inhibited kynurenine production, increased intracellular Na^+^ levels, and downregulated IDO1 mRNA and protein levels. These compounds decreased IDO1 expression through suppression of signal transducers and activators of transcription 1 (STAT1) activation, indicating that cardiac glycosides may regulate immune checkpoint proteins by targeting STAT1 (Shandell et al., 2022).

**FIGURE 2:**
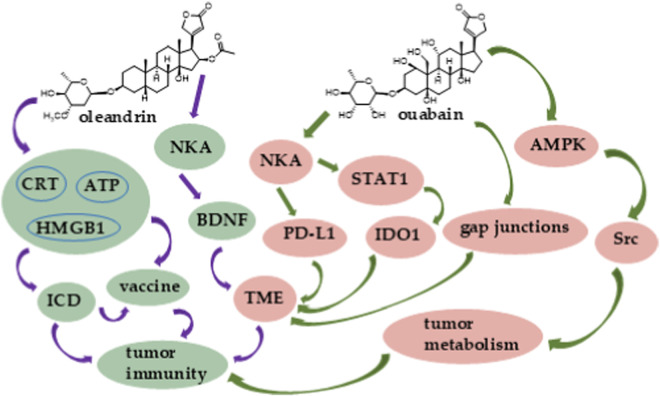
Major connections between tumor immunity and oleandrin (left) or ouabain (right).

The cardiac glycoside digoxin, a well-known therapeutic agent isolated originally from *Digitalis lanata* Ehrh. (Plantaginaceae), has long been used for the treatment of heart failure and atrial fibrillation. It also binds to NKA and inhibits its activity and targets other proteins and signaling pathways to show potential anticancer activity (Ren et al., 2020; Ren et al., 2021; Ren et al., 2024). Interestingly, digoxin was one of the compounds that were identified for the first time to affect the function of retinoic acid-related orphan receptor γ T (RORγT), a protein expressed mainly in T helper 17 (Th17) cells. Digoxin induces RORγ/RORγT-dependent transcription in human HepG2 liver cancer and Th17 lymphocytes. Of these, Th17 cells are involved in cancer and several autoimmunological diseases and have been regarded as promising targets for adoptive cell therapy (ACT) (Figure 1). As a potent RORγ/RORγT receptor activator, digoxin may be able to be used potentially in the design and development of ACT (Karas et al., 2019). In addition, digoxin targets STAT1 to decrease the immune checkpoint protein IDO1 expression in human lung and breast cancer cells (Figure 2) (Shandell et al., 2022).

A combination of digoxin and P-cis (a co-polymer of cisplatin) induced calreticulin (CRT) exposure and triphosadenine (adenosine triphosphate, ATP) secretion and elevated high mobility group box 1 (HMGB1) release and thus induced ICD (Figure 1). As a result, DC maturation was promoted, with the CD8^+^ CTL response being activated. Cancer cells treated by this combination of digoxin and P-cis were prepared as vaccines intended to prevent tumor recurrence and metastasis (Xiang et al., 2020). Hence, in this manner, digoxin may exhibit a vaccine-like function for the improvement of cancer immunotherapy.

The cardiac glycoside oleandrin is the major active component of the toxic shrub, *Nerium indicum* L. (Apocynaceae) and exhibits potential anticancer activity (Ren et al., 2024). It stimulates neurons to release the brain-derived neurotrophic factor (BDNF) to reduce tumor cell chemotaxis and to modulate the TME and thus has shown antitumor potential against gliomas (Garofalo et al., 2017). In CT26 mouse colon cancer cells, oleandrin induced late cellular apoptosis and necrosis, expression of CRT, and secretion of ATP and HMGB1, and thus activated ICD. A dual controlled release microsphere/hydrogel platform developed for oleandrin, namely, OL-M/Gel, showed potential effects on tumor immunity. OL-M/Gel induced ICD to suppress tumor growth, and it controlled precisely the output of oleandrin in tumors to improve its antitumor efficacy, with no significant side effects being observed (Figure 2) (Gao et al., 2023). Another important cardiac glycoside, ouabain, was isolated from *Acokanthera schimperi* (A. DC.) Benth. & Hook. f. (Apocynaceae), which blocks NKA and has been used traditionally for the treatment of cardiac dysfunctions and shows anticancer potential through its effects on the inflammatory immune responses and the TME (Matozzo et al., 2020; Ren et al., 2024). Functioning as an immunomodulator, ouabain was found to regulate lymphocyte proliferation, cytokine production, and monocyte functions (Rodrigues-Mascarenhas et al., 2009; Yin et al., 2015). Interestingly, ouabain also promoted gap junctional intercellular communication (GJIC) in cancer cells and activated the AMPK-Src signaling pathways to altered cancer cell metabolism (Serrano-Rubi et al., 2020; Shen et al., 2020) (Figure 2). Cancer cells can avoid mitochondrial activity and oxidative phosphorylation and rely on glycolysis to produce energy. This specific metabolism helps cancer cells avoid immune attacks and produce several chemicals to alter immune functions (Villalba et al., 2013). Furthermore, ouabain decreased the expression of the immune checkpoint protein, IDO1, by targeting STAT1 (Figure 2) (Shandell et al., 2022). The endogenous ouabain also could reprogram tumor immunity by regulating PD-L1 expression (Yang et al., 2023), indicating that this cardiac glycoside could be used potentially to support the effectiveness of other cancer immunotherapeutic agents.

## Concluding remarks and discussion

Cancer immunotherapy has proved as an effective treatment of cancer, but several challenges have been reported, including immune evasion in the TME and limit effectiveness for the treatment of certain solid tumors (Ma et al., 2025). As discussed above in the present perspective article, ion channels and pumps regulate cellular processes and thus are important for the proliferation of cancer cells and for activities and functions of immune cells. NKA correlates with ion channels and H^+^-ATPase and shows effects on tumor immunity, and its inhibitors, cardiac glycosides, such as digoxin, oleandrin, and ouabain, may be used potentially in a combination strategy to support cancer immunotherapy.

A raised Na^+^ level due to disrupted Na^+^ homeostasis affects cell volume and metabolism (Leslie et al., 2019). Interestingly, the endogenous ouabain increases transcription of PD-L1 (Yang et al., 2023), while the non-pumping NKA is involved in ouabain-induced VRAC activation (Fujii et al., 2018). Also, this cardiac glycoside was found to promote GJIC and to activate the AMPK-Src signaling pathways to altered cancer cell metabolism (Serrano-Rubi et al., 2020; Shen et al., 2020). Thus, cardiac glycosides may target the TME through the regulation of the cell volumes and metabolism and cellular communications to be supportive of the tumor immunity.

Immunogenic cell death (ICD) can induce an effective antitumor immune response through the activation of dendritic cells (DCs) and the resultant T cell response. It is characterized by the release of molecular signals, which are in general referred to damage-associated molecular patterns (DAMPs), including CRT translocation and ATP and HMGB1 secretion. These DAMPs can further induce DC maturation, DC-mediated tumor antigen cross-presentation, and T-cell polarization. Thus, the dying cancer cells initiate a robust immune response, acting as an “anticancer vaccine” (Gao et al., 2023; Krysko et al., 2012). Both digoxin and oleandrin can induce ICD, indicating that these cardiac glycosides may have potential for the use in the development of cancer vaccines.

**SCHEME 1:**
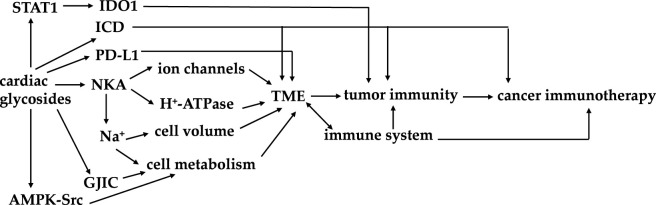
Signaling pathways for cardiac glycosides to mediate their potential on cancer immunotherapy.

Cardiac glycosides show their potential effects on tumor immunity mainly through their interaction with NKA and the related signaling pathways, their property on AMPK-Src, GJIC, PD-L1, and STAT1 signaling pathways, and their induction of ICD (Scheme 1). Of these, NKA restores membrane Na^+^ and K^+^ electrochemical gradients and thus correlates with ion channels and H^+^-ATPase to mediate its regulatory effects on immune response (Buch-Pedersen et al., 2006; Li et al., 2006). PD-1 is a co-inhibitory receptor expressed in immune cells, of which the ligand PD-L1 acts as an immune checkpoint and is overexpressed in cancer cells to contribute to cancer immune evasion. PD-L1 can be regulated by the IFN-γ-JAK-STAT signaling pathway, for which IFN-γ induces the PD-L1 expression, and JAK and STAT play an essential role. Thus, PD-L1 inhibitors can support T cells to recognize and destroy cancer cells through PD-1/PD-L1 blockade (Zhang et al., 2018).

Thus, NKA could be a potential target for tumor immunity, for which its inhibitors, cardiac glycosides, may have potential use. However, NKA is non-selectively expressed in nearly all types of cells, indicating that inhibition of this enzyme may result in toxicity or serious side effects, as indicated by the toxicity observed for several cardiac glycosides. Thus, synthetic modification of these agents by focusing their interaction with NKA could be a promising approach to decrease their toxicity and to increase their effects on tumor immunity.

## Data Availability

The original contributions presented in the study are included in the article/supplementary material, further inquiries can be directed to the corresponding authors.

## Glossary

a2V: V-ATPase a2 isoform
ACT: Adoptive cell therapy
APC: Antigen-presenting cell
ASIC: Acid-sensing ion channel
ATP: Adenosine triphosphate
BDNF: Brain-derived neurotrophic factor
CACC: Calcium-activated chloride channel
CAR: Chimeric antigen receptor
CCL: C-C chemokine ligand
cGAS: Cyclic guanosine monophosphate–adenosine monophosphate synthase
circRNAs: Circular RNAs
CRAC: Ca^2+^ release-activated Ca^2+^
CRT: Calreticulin
CSC: Cancer stem cell
CTL: Cytotoxic T lymphocyte
DAMP: Damage-associated molecular pattern
DC: Dendritic cell
GJIC: Gap junctional intercellular communication
HMGB1: High mobility group box 1
ICB: Immune checkpoint blockade
ICD: Immunogenic cell death
IDO1: Indoleamine-pyrrole 2′,3′-dioxygenase 1
IFN: Interferon
IL: Interleukin
**K**_**ATP**_: ATP-sensitive K^+^
KCa: Calcium-activated K^+^
LRRC8: Leucine-rich repeat-containing protein 8
mTOR: Mammalian target of rapamycin
NF-κB: Nuclear factor κ-light-chain-enhancer of activated B cells
NK: Natural killer
NKA: Na^+^/K^+^-ATPase
NSCLC: Non-small cell lung cancer
PD-1: Programmed death receptor 1
PD-L1: Programmed death ligand 1
Piezo1: Piezo-type mechanosensitive ion channel component 1
PSCA: Prostate stem cell antigen
RORγ: Retinoic acid-related orphan receptor γ
SOC: Store-operated Ca^2+^
SOCE: Store-operated Ca^2+^ entry
STAT1: Signal transducers and activators of transcription 1
STIM1: Stromal interaction molecule 1
STING: Stimulator of interferon genes
TAN: Tumor associated neutrophil
TCR: T cell receptor
Th: T-helper
TME: Tumor microenvironment
TMEM16: Transmembrane protein 16
V-ATPase: Vacuolar H^+^-ATPase
VGCC: Voltage-gated Ca^2+^ channel
VRAC: Volume-regulated anion channel
